# Racial and Ethnic Representation in Food Allergen Immunotherapy Trial Participants: A Systematic Review

**DOI:** 10.1001/jamanetworkopen.2024.32710

**Published:** 2024-09-16

**Authors:** Hannah Suffian, Aarti Pandya, Lauren Davidson, Vincent Staggs, Bridgette L. Jones

**Affiliations:** 1 University of Missouri-Kansas City School of Medicine, Kansas City; 2 Department of Pediatrics, University of Missouri-Kansas City School of Medicine, Kansas City; 3 Children’s Mercy Hospital, University of Missouri-Kansas City School of Medicine, Kansas City; 4 Section of Allergy/Immunology, University of Missouri-Kansas City School of Medicine, Kansas City; 5 Division of Health Services and Outcomes Research, University of Missouri-Kansas City School of Medicine, Kansas City; 6 Division of Pediatric Clinical Pharmacology and Therapeutic Innovation, University of Missouri-Kansas City School of Medicine, Kansas City

## Abstract

**Importance:**

The lack of inclusion of diverse population samples in food allergy immunotherapy clinical trials not only leads to decreased applicability to the general population in terms of results and treatments but can also be seen as a broader social injustice contributing to inequity within the health care system.

**Objectives:**

To investigate the racial and ethnic distribution of participants included in food allergy immunotherapy clinical trials, and determine whether the racial and ethnic representation in trials accurately reflects the patients who experience food allergy.

**Evidence Review:**

Data were collected from articles found on PubMed and ClinicalTrials.gov using key terms of *food hypersensitivity*, *food allergy*, and *immunotherapy*, while also incorporating specific criteria such as clinical trials conducted within the last 5 years with children aged from birth to 18 years old. Articles were selected based on their relevance to the research question. Main outcomes were totals and percentages of trial participants by race and ethnicity, stratified by pediatric trials, site of study, and National Institutes of Health funding.

**Findings:**

Thirty-five articles were initially identified, of which 34 were classified as human clinical trials. Of these trials, 26 met criteria of an original randomized clinical trial and included racial and ethnic demographics for analysis in the study. Among trials included, the majority of the 3689 participants identified as White (2640 participants [72.0%]), followed by Black or African American (293 participants [8.0%]), Asian (239 participants [6.0%]), multiple races or other (210 participants [6.0%]), Hispanic or Latino (96 participants [3.0%]), American Indian (3 participants [<1.0%]), and Native American or Pacific Islander (3 participants [<1.0%]). We observed differences in racial and ethnic inclusion by study site (US vs external to US) and funding support (National Institutes of Health vs industry or other non–National Institutes of Health sources).

**Conclusions and Relevance:**

In this systematic review of racial and ethnic diversity in food allergy immunotherapy trials, there was a lack of diversity relative to the overall food allergy burden among Black and Hispanic patients, indicating important gaps in the conduct of pediatric clinical trials, especially for treatments that are meant for use in broad populations where significant race- and ethnicity-related disparities exist. Working to correct this disparity will not only increase the usefulness of future clinical trial data but can further assist in alleviating public health inequities.

## Key Points

**Question:**

What are the racial and ethnic distributions observed among participants in food allergy immunotherapy trials?

**Findings:**

In this systematic review of 26 randomized clinical trials, the predominant racial demographic group represented within food allergy immunotherapy clinical trials was White. In contrast, Black and Hispanic participants were underrepresented despite an increased disease burden in these populations.

**Meaning:**

The underrepresentation of Black and Hispanic participants in food allergy clinical trials raises concerns about the generalizability and applicability of clinical trial results among these highly burdened populations.

## Teaser

This systematic review of food allergy immunotherapy clinical trials examines representation of racial and ethnic minority groups given the increased disease burden of these populations.

## Introduction

It is estimated that 5.6% of children in the US are affected by food allergy^1^; food allergy is the most common cause of anaphylaxis in both children and adults.^2^ Food allergy management has evolved over the last few years to include food immunotherapy as a treatment option for food desensitization and prevention of anaphylaxis. Arachis hypogaea powder is the first food immunotherapy product approved by the US Food and Drug Administration (FDA), and products for other food allergens (egg,^3^ milk,^4^ tree nut,^5^ sesame^6^) have been studied in clinical trials.

Racial and ethnic disparities in food allergy prevalence, diagnosis, treatment, and outcomes have been well documented. Children identifying as Black and/or Hispanic have higher odds of diagnosis for multiple food allergies in comparison with children who identify as non-Hispanic White, and Black Americans experience a greater burden of food-allergic disease and complications when compared with White Americans.^7^ In a birth cohort study, children who identified as Black were found 3-times more likely than other children to have peanut immunoglobulin E (IgE) higher than 95% of the predictive decision point for peanut allergy diagnosis.^8^ Similarly, it has been found that prevalence of food allergy in adults is higher among all ethnic and racial groups other than White.^9^ Food allergies add psychological stress on patients and caregivers and cause increased financial strain from costs of allergen-free foods. These burdens are compounded in already marginalized and oppressed populations. As such, food allergy interventions are important to closing disparity gaps and should be expected to include these highly affected populations.

In 2022, the FDA published guidelines to inform industry sponsors on the development of plans to increase enrollment of participants from racial and ethnic backgrounds that are underrepresented in clinical trials.^10^ Dr Janet Woodcock, then FDA commissioner, stated “physicians’ ability to extrapolate from trial results to their own patients would be dramatically improved if a trial’s participants reflected the product’s intended patient population as accurately as possible,” and described 2020 FDA data indicating lack of racial and ethnic inclusion among industry-sponsored clinical trials.^11^ We have previously described lack of inclusion among clinical trials of monoclonal antibody therapies in children and suspect that similar underrepresentation is observed in food allergy trials.^12^

In the study reported here, we aimed to describe the racial and ethnic distribution of participants included in food allergy immunotherapy trials based on our hypothesis that disparities exist in the racial and ethnic representation among food allergy clinical trials. While we discuss food allergy in children and adults, attention is focused on the pediatric population as the prevalence of food is highest among this group. This information may provide perspective for improvements needed in food allergy clinical trial design and implementation to assure generalizability of results and effectiveness of treatment.

## Methods

To identify trials for inclusion in our analysis we conducted a PubMed search with professional librarian services support from our School of Medicine (eFigure in Supplement 1). The search used the key terms *food hypersensitivity*, *desensitization*, *food allergy*, and *immunotherapy*, while also incorporating specific criteria such as clinical trials conducted within the last 5 to 10 years and studies with inclusion of children aged from birth to 18 years old. A similar search was conducted within ClinicalTrials.gov using the criteria of food allergy in children, from birth to age 17 years, with the key terms *immunotherapy* and *desensitization*. While focusing our search on the pediatric population, many trials also evaluated food allergy in adults, which we included as well. The search was started and completed during July 2022. Articles were then evaluated and selected based on their relevance to the research question, whether they included race and ethnicity data, and their status as a randomized clinical trial. To facilitate data collection and analysis, the counts of participants were grouped according to race and ethnicity, age (stratified as ages 0-18 years and 19 years and older), location of the trial (conducted in the US or outside the US or both), and whether the trial was funded by the NIH or industry supported. Race and ethnicity frequencies as reported in the included studies and ClinicalTrials.gov data were calculated by trial age group, location, and funder. This report follows the Preferred Reporting Items for Systematic Reviews and Meta-analyses (PRISMA) reporting guideline.

## Results

Forty-six articles were initially identified, of which 35 were classified as human clinical trials. Of these trials, 26 studies (with 3689 participants) met criteria for an original randomized clinical trial and included racial and ethnic demographics for analysis in the study (Table 1). The following racial and ethnic distribution was observed across all included trials reporting data on participants race and ethnicity: 3 American Indian (<0.1%), 239 Asian (6.0%), 293 Black or African American (8.0%), 96 Hispanic or Latino (3.0%), White (2640 [72.0%]), 3 identifying as other Native American or Pacific Islander (<0.1%), and 210 identifying as multiple races or other (210 [6.0%]) (Table 2).

**Table 1.** Clinical Trials Reporting Racial and Ethnic Demographics

Study	Trial name	Participants, No. (%)^a^						
		White	Black or African American	American Indian	Hispanic	Asian	Native American or Pacific Islander	Other or multiple races
Bird et al,^13^ 2018	Efficacy and safety of AR101 in Oral immunotherapy for peanut allergy: Results of ARC001, a randomized, double-blind, placebo-controlled phase 2 clinical trial	46 (83.6)	3 (5.5)	1 (1.8)	NA	2 (3.6)	0	3 (5.5)
Vickery et al,^14^ 2017	Early oral immunotherapy in peanut-allergic preschool children is safe and highly effective	33 (89.2)	3 (8.1)	NA	NA	NA	NA	1 (2.7)
Chinthrajah et al,^15^ 2019	Sustained outcomes in oral immunotherapy for peanut allergy (POISED study): A large, randomized, double-blind, placebo-controlled, phase 2 study	74 (60.2)	2 (1.6)	NA	3 (2.4)	32 (26.0)	1 (0.8)	11 (8.9)
Andorf et al,^16^ 2018	Anti-IgE treatment with oral immunotherapy in multifood allergic participants: A double-blind, randomized, controlled trial	0	0	0	3 (6.3)	0	0	45 (93.8)
Wood et al,^17^ 2016	A randomized, double-blind, placebo-controlled study of omalizumab combined with oral immunotherapy for the treatment of cow’s milk allergy	49 (86.0)	NA	NA	NA	NA	NA	NA
Jones et al,^18^ 2016	Safety of epicutaneous immunotherapy for the treatment of peanut allergy: A phase 1 study using the viaskin patch	78 (73.6)	19 (17.9)	NA	6 (5.7)	1 (0.9)	NA	2 (1.9)
Metcalfe et al,^19^ 2016	Elevated IL-5 and IL-13 responses to egg proteins predate the introduction of egg in solid foods in infants with eczema	52 (76.5)^b^	NA	NA	NA	NA	NA	16 (23.5)^b^
Palmer et al,^20^ 2013	Early regular egg exposure in infants with eczema: A randomized controlled trial	68 (79.1)^b^	NA	NA	NA	NA	NA	18 (20.9)^b^
Varshney et al,^21^ 2011	A randomized controlled study of peanut oral immunotherapy: clinical desensitization and modulation of the allergic response	27 (96.4)	NA	NA	NA	NA	NA	1 (3.6)
Kim et al,^22^ 2011	Sublingual immunotherapy for peanut allergy: Clinical and immunologic evidence of desensitization	17 (94.4)	NA	NA	NA	1 (5.6)	NA	NA
Burks et al,^23^ 2015	Sublingual immunotherapy for peanut allergy: Long-term follow-up of a randomized multicenter trial	36 (90.0)	1 (2.5)	NA	NA	3 (7.5)	NA	NA
NCT01846208	Baked egg or egg oral immunotherapy for children with egg allergy (CoFAR7)	72 (79.1)	3 (3.3)	0	2 (2.2)	8 (8.8)	0	6 (6.6)
NCT00597727	A study of sublingual immunotherapy in peanut-allergic children (SLB)	54 (90.0)	1 (1.7)	0	0	4 (6.7)	0	1 (1.7)
NCT01373242	Sublingual immunotherapy for peanut allergy and induction of tolerance (SLIT-TLC)	49 (90.7)	0	0	0	4 (7.4)	0	1 (1.9)
NCT00461097	Oral immunotherapy for childhood egg allergy	48 (82.8)	2 (3.4)	0	3 (5.2)	5 (8.6)	0	0
NCT02304991	FARE peanut SLIT and early tolerance induction (FARE/SLIT)	44 (86.3)	2 (3.9)	1 (2.0)	NA	1 (2.0)	1 (2.0)	2 (3.9)
NCT01867671	Peanut oral immunotherapy in children (IMPACT)	95 (62.5)	6 (3.9)	0	6 (3.9)	18 (11.8)	0	27 (17.8)
NCT02635776	Peanut allergy oral immunotherapy study of AR101 for desensitization in children and adults (PALISADE) (PALISADE)	438 (73.2)	9 (1.5)	1 (0.2)	47 (7.9)	54 (9.0)	1 (0.2)	0
NCT03201003	ARTEMIS peanut allergy in children (ARTEMIS)	140 (79.1)	0	0	2 (1.1)	2 (1.1)	0	3 (1.7)
NCT02636699	Efficacy and safety of viaskin peanut in children with Immunoglobulin E (IgE)-mediated peanut allergy (PEPITES)	290 (81.5)	3 (0.8)	NA	NA	27 (7.6)	NA	36 (10.1)
Jones et al,^24^ 2016; NCT01904604	Peanut epicutaneous phase II immunotherapy clinical trial	63 (80.8)	3 (3.8)	0	4 (5.1)	2 (2.6)	0	6 (7.7)
NCT01675882	Efficacy and safety of several doses of viaskin peanut in adults and children with peanut allergy (VIPES)	135 (61.1)	5 (2.3)	0	3 (1.4)	27 (12.2)	0	14 (6.3)
Scurlock et al,^25^ 2021	Epicutaneous immunotherapy for treatment of peanut allergy: Follow-up from the Consortium for Food Allergy Research	59 (85.5)	NA	NA	NA	NA	NA	10 (14.5)
Holl et al,^26^ 2020	A randomized trial of the acceptability of a daily multi-allergen food supplement for infants	360 (51.1)	219 (31.1)	NA	78 (11.1)	NA	NA	NA
Pongracic et al,^27^ 2022	Safety of epicutaneous immunotherapy in peanut-allergic children: REALIZE randomized clinical trial results	292 (74.3)	8 (2.0)	NA	9 (2.3)	45 (11.5)	NA	39 (9.9)
NCT03462030	Efficacy and safety of baked milk oral immunotherapy in children with severe milk allergy: A randomized, double-blind, placebo-controlled phase 2 trial	21 (67.7)	4 (12.9)	0	1 (3.2)	3 (9.7)	0	2 (6.5)

Abbreviation: NA, not applicable.

^a^ NA indicates the trial did not include that racial or ethnic category.

^b^ Reported demographics in the trial are that of mother, not child participant.

**Table 2.** Overall Racial and Ethnic Demographics Included in Food Allergy Immunotherapy Trials

Racial or ethnic category	Participants, No. (%)						
	All (N = 3689)	Pediatric only (n = 2399)	Pediatric and adults (n = 1290)	US only (n = 2967)	Not US only (n = 722)	NIH-funded (n = 1397)	Not NIH-funded (n = 2292)
Total trials, No.	26	17	9	22	4	17	9
American Indian	3 (<0.1)	1 (<0.1)	2 (0.2)	3 (0.1)	0	2 (<0.1)	1 (<0.1)
Asian	239 (6.5)	118 (4.9)	121 (9.4)	192 (6.5)	47 (6.5)	125 (8.9)	114 (5.0)
Black	293 (7.9)	251 (10.5)	42 (3.3)	285 (9.6)	8 (1.1)	34 (2.4)	259 (11.3)
Hispanic (race and ethnicity category)^a^	96 (2.6)	90 (3.8)	6 (0.5)	87 (2.9)	9 (1.2)	15 (1.1)	81 (3.5)
Hispanic (separate variable)^b^	71 (1.9)	14 (0.6)	57 (4.4)	69 (2.3)	2 (0.3)	15 (1.1)	56 (2.4)
Native American or Pacific Islander	3 (<0.1)	1 (<0.1)	2 (0.2)	3 (0.1)	0	2 (0.1)	1 (<0.1)
Non-White^c^	34 (0.9)	34 (1.4)	0	0	34 (4.7)	0	34 (1.5)
Other or multiple	210 (5.7)	164 (6.8)	46 (3.6)	168 (5.7)	42 (5.8)	153 (11.0)	57 (2.5)
Unknown	171 (4.6)	78 (3.2)	93 (7.2)	141 (4.8)	30 (4.2)	8 (0.6)	163 (7.1)
White	2640 (71.6)	1662 (69.3)	978 (75.8)	2088 (70.4)	552 (76.5)	1058 (75.7)	1582 (69.0)

Abbreviation: NIH, National Institutes of Health.

^a^ Count of patients identified as Hispanic in studies with Hispanic as one of several race and ethnicity categories.

^b^ Count of patients identified as Hispanic in studies with a separate variable for Hispanic ethnicity (rather than Hispanic as one of several race and ethnicity categories).

^c^ No other information given in response.

### Racial and Ethnic Distribution Among US Studies

**Figure 1.**
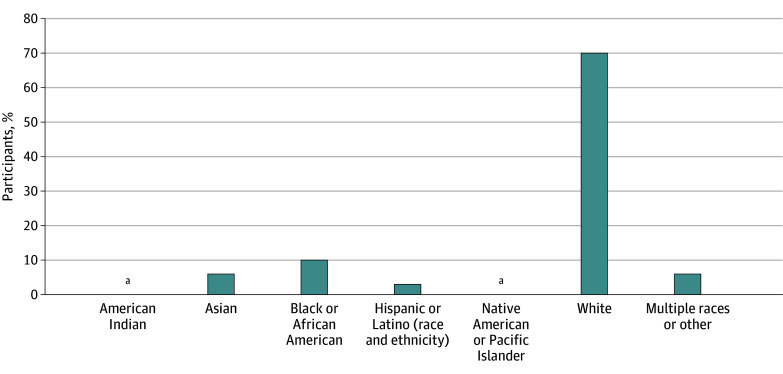
Racial and Ethnic Distribution Included in US Clinical Trials ^a^3 participants.

**Figure 2.**
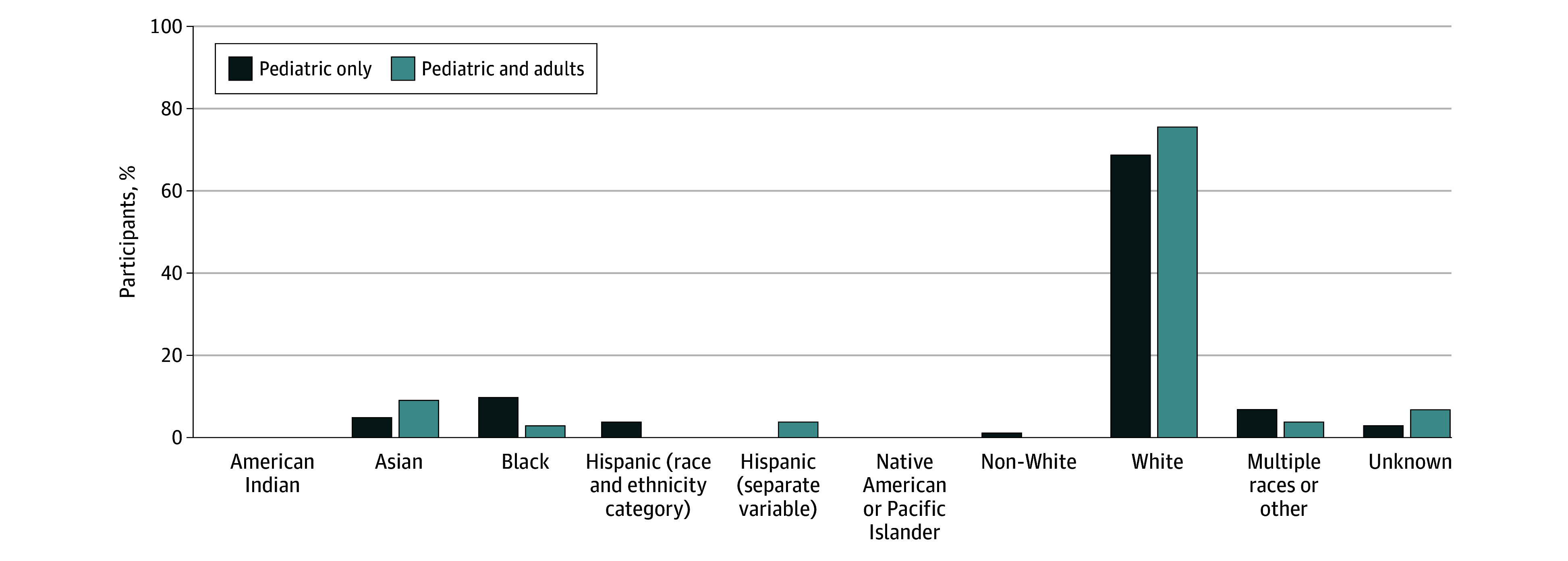
Racial and Ethnic Distribution Included in US Clinical Trials by Pediatric and Adult Participants

Studies conducted in the US included 3 American Indian participants (<0.1%), 192 Asian participants (6.0%), 285 Black or African American participants (10.0%), 87 Hispanic or Latino participants (3.0%), 2088 White (70.0%)3 identifying as other Native American or Pacific Islander (<0.1%), and 168 identifying as multiple races or other (6.0%) (Figure 1). When comparing trials conducted in pediatric only vs combined pediatric and adult populations, some differences did exist. Black participants made up 10% of those included within pediatric-only studies in comparison with 3% in combined pediatric and adult studies. In contrast, Asian participants made up 9% of participants in combined pediatric and adult studies vs 5% in pediatric-only studies (Figure 2).

### Trials Funded by the NIH

**Figure 3.**
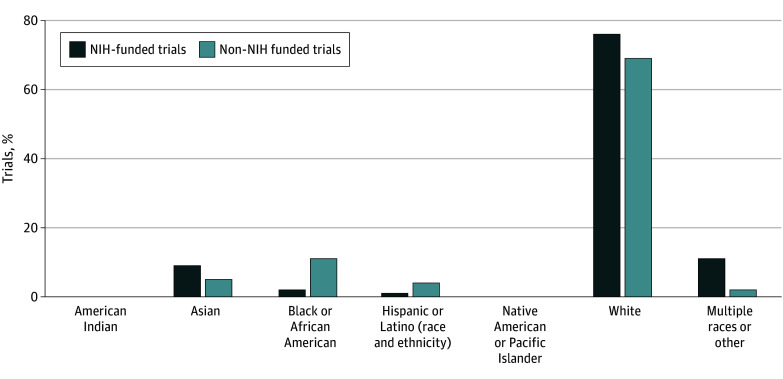
Comparison of Racial and Ethnic Demographics of Participants Included in NIH-Funded vs Non-NIH Funded Trials

For trials funded by the NIH, the racial and ethnic distribution was 2 American Indian participants (<0.1%), 125 Asian (9.0%), 34 Black or African American (2.0%), 15 Hispanic or Latino (1.0%), 1058 White (76.0%), 2 identifying as other Native American or Pacific Islander (<0.1%), and 153 identifying as multiple races or other (11.0%) (Figure 2). For comparison, in studies not funded by the NIH there was 1 American Indian (both <0.1%), 114 Asian (5.0% vs 9.0%), 259 Black or African American (11.0% vs 2.0%), 81 Hispanic or Latino (4.0% vs 1.0%), 1582 White (69.0% vs 76.0%), 1 identifying as Native American or Pacific Islander (both <0.1%), and 57 identifying as multiple races or other (2.0% vs 11.0%) (Figure 3).

## Discussion

In this study, pediatric and adult food immunotherapy clinical trials included predominantly White-identifying participants. Participants identifying as Black or Hispanic were markedly underrepresented in comparison with the general US population. Among pediatric only trials, there was increased representation of those identifying as Black and/or Hispanic. However, there was still underrepresentation compared with the general population of children in the US.

Racial and ethnic representation within NIH- vs non-NIH funded trials included largely White participants with less representation from Black and Hispanic participants compared with non-NIH funded studies. Black participants made up only 2% of NIH-funded trials but 11% in non-NIH funded studies. According to NIH policy, the Public Health Service Act sec. 492B, 42 USC. sec. 289a-2 ensures the inclusion of members of racial and ethnic minority groups in all NIH-funded clinical research in a manner that is appropriate to the scientific question under study. The NIH describes that this mandate as intended to support findings that are generalizable to the general population. The policy states “the statute requires clinical trials to be designed to analyze whether study outcomes differ for members of racial and ethnic minority groups.”^28^ Powering such an analysis would require enough participants to be included from relevant racial and ethnic groups.

The NIH enforces this mandate by requiring researchers to submit inclusion plans within a human participants section of the grant application describing the intended participant population for the proposed study. Investigators also must justify reasons for exclusion of specific populations. Inclusion plans are vetted within the grant review process by peer reviewers and determined to be acceptable or unacceptable. Additional checkpoints are implemented in the postaward period, when researchers are required to report information on race and ethnicity of enrolled participants in annual progress reports for review by NIH.^28^ Our findings demonstrate that these methods to ensure inclusion and generalizability may not be effective. Gaps in this strategy include the lack of a specific, designated benchmark that peer reviewers use to determine whether inclusion plans are acceptable. Likewise, it is not clear that NIH follows any specific guidance when reviewing enrollment data. Strategies to ensure that study populations reflect not only the US general population but those most affected by the studied disease or condition are needed for development of clinical trials that yield data generalizable across the population and especially to those most affected by a given condition.

Non-NIH funded trials appeared to have increased racial diversity, a surprising finding as academic centers where most NIH-funded studies are conducted typically serve a racially diverse population. It might be expected that NIH trials would better reflect the general population and those impacted by food allergy, but industry trials often conduct participant enrollment across multiple study sites, which can lead to increased diversity. Prior to the 2022 FDA guidance to inform the development of diversity plans for clinical trial inclusion, there was no specific guidance or requirement to include participants from specific demographic subgroups in clinical trials. Sponsors were only required to submit data on enrollment (if any) from such subgroups, such as racial and ethnic groups, in annual reports and to conduct analysis of data by subgroup.^29^ Despite having increased racial and ethnic diversity in comparison with NIH-funded trials, non-NIH trials were not representative of the food allergy patient population, indicating the necessity of diversity plans for enrollment.

Lack of racial and ethnic diversity among trials related to allergy and asthma has been previously described.^12,30^ However, this is the first study, to our knowledge, to describe the makeup of trials related to food allergy immunotherapy. We believe that our findings are especially important to highlight, as this disparity will likely influence policy decisions that would determine who is able to benefit from food allergy immunotherapy treatments. Racial and ethnic groups may exhibit differences in the clinical response and uptake of therapeutics due to the influence of underlying sociocultural and environmental factors that drive disease and influence acceptability of interventions. Recently, studies have described differences in treatment response to inhaled corticosteroids for children who identified as Black relative to those identifying as White and suggested a superior response to inhaled steroids among Black children.^31^ Racial group classification is understood as a social construct, and it is understood that race is not aligned with inherent biological differences. However, exposures related to interpersonal (eg, chronic stress), structural (eg, bias in medicine), and institutional (eg, wealth inequity) racism has been shown to affect biological mechanisms (eg, epigenetic changes) that may contribute to underlying disease endotype and exacerbate potential differences in the effectiveness of therapeutics and other interventions. Clinical trial inclusion is critical to the generalizability of effective therapies and interventions.

It is essential that actionable goals are made to ensure that clinical trials include a participant population that is representative of the patient population. Such action is needed to improve the efficacy, safety, and effectiveness of novel therapies in the general population and may be especially important in closing disparity gaps. Meaningful racial and ethnic diversity in clinical trial enrollment can be achieved through a variety of measures, such as (1) established, predefined goals and metrics to meet statistical powering, (2) predefined targeted recruitment and retention strategies for underrepresented populations relevant to the disease or condition, (3) participant-friendly study designs that accommodate barriers and needs of participants, (4) the incorporation of language services for multilingual study documents and participant resources, (5) sustained outreach and engagement with community groups that represent underrepresented groups for current and future research, (6) mutually beneficial capacity building within community engaged research activities, (7) improved relationships between research institutions and historically marginalized groups to foster trust, (8) workforce development to increase representation of researchers from underrepresented groups and diverse research staff included those with multilingual skillsets, (9) implementation of research and science pathway programs so that local children and communities can directly benefit from the research enterprise, (10) funding to test and develop effective strategies for recruitment and engagement of those underrepresented in research, and (11) accountability by funding and governmental agencies to ensure appropriate racial and ethnic inclusion in clinical trials.

### Limitations

Limitations of our study include that the method of race and ethnicity determination (eg, self-report vs investigator determined) varied across studies and was not always discussed in the papers included. Therefore, race and ethnicity classification may not have been always accurate. Furthermore, the race and ethnicity classification groups varied across studies. For example, in some studies Hispanic ethnicity was utilized to classify both a race and ethnicity group and in other studies participants were categorized as Hispanic ethnicity type (Hispanic vs non-Hispanic). In other studies ethnicity was not included at all as a demographic category, and one may assume that Hispanic participants were included within the described racial groups. This variability limits comparisons across studies and leads to misclassification bias, where in some cases one may conclude that a particular ethnic or racial group was not included when in fact they were but not described in the data.

## Conclusions

Lack of meaningful inclusion of racial and ethnic groups in clinical trials not only limits the potential benefits and effectiveness of health care interventions but also compounds the broader issue of social injustice and inequity in health care. By ensuring diversity and inclusivity in clinical trials, we can both improve health care outcomes and help increase equity and justice in health care.

## Notes

Supplement 1. **eFigure.** PRISMA 2020 Flow Diagram for New Systematic Reviews Which Included Searches of Databases, Registers, and Other Sources

Supplement 2.
Data Sharing Statement
