# Supplementary material for: Racial and Ethnic Representation in Food Allergen Immunotherapy Trial Participants: A Systematic Review
Source: JAMA Netw Open. 2024 Sep 16;7(9):e2432710. doi: 10.1001/jamanetworkopen.2024.32710 (PMC11406388; doi:10.1001/jamanetworkopen.2024.32710)
Supplement: Supplement 1. — eFigure. PRISMA 2020 Flow Diagram for New Systematic Reviews Which Included Searches of Databases, Registers, and Other Sources [file jamanetwopen-e2432710-s001.pdf]

Supplemental Online Content

Suffian H, Pandya A, Davidson L, Staggs V, Jones BL. Racial and ethnic representation in food allergen immunotherapy trial participants. *JAMA Netw Open.* 2024;7(9):e2432710. doi:10.1001/jamanetworkopen.2024.32710

eFigure. PRISMA 2020 Flow Diagram for New Systematic Reviews Which Included Searches of Databases, Registers, and Other Sources

This supplemental material has been provided by the authors to give readers additional information about their work.

© 2024 Suffian H et al. *JAMA Network Open.*

eFigure. PRISMA 2020 flow diagram for new systematic reviews which included searches of databases, registers and other sources

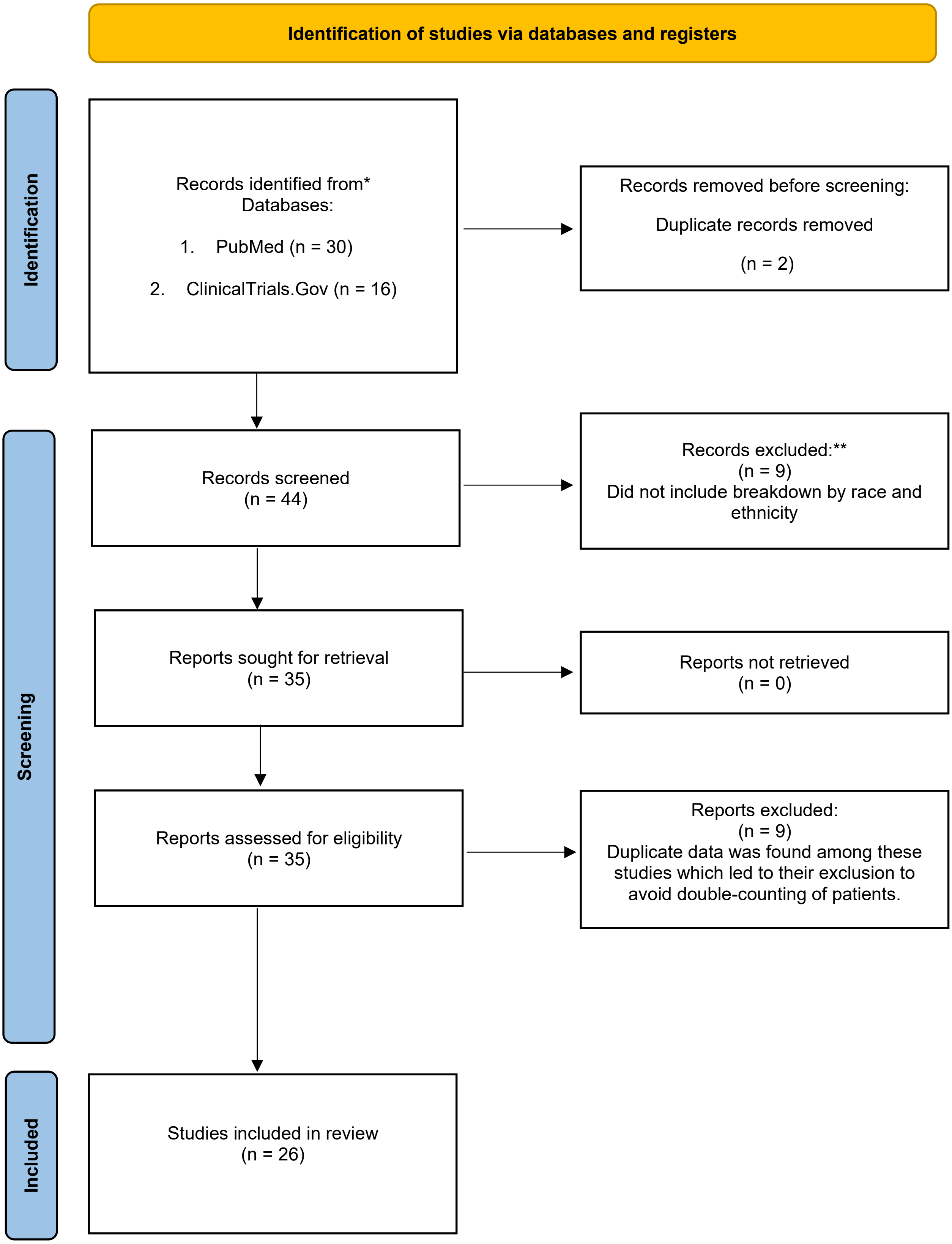

*Consider, if feasible to do so, reporting the number of records identified from each database or register searched (rather than the total number across all databases/registers).
**If automation tools were used, indicate how many records were excluded by a human and how many were excluded by automation tools.

From: Page MJ, McKenzie JE, Bossuyt PM, Boutron I, Hoffmann TC, Mulrow CD, et al. The PRISMA 2020 statement: an updated guideline for reporting systematic reviews. BMJ 2021;372:n71. doi: 10.1136/bmj.n71. For more information, visit: http://www.prisma-statement.org/

© 2024 Suffian H et al. *JAMA Network Open.*
